# The Influence of Induced Emotions on Distance and Size Perception and on the Grip Scaling During Grasping

**DOI:** 10.3389/fpsyg.2021.651885

**Published:** 2021-09-28

**Authors:** Chuyang Sun, Juan Chen, Yuting Chen, Rixin Tang

**Affiliations:** ^1^Department of Psychology, School of Social and Behavioral Sciences, Nanjing University, Nanjing, China; ^2^Key Laboratory of Brain, Cognition and Education Sciences (South China Normal University), Ministry of Education, China; ^3^School of Psychology, Center for Studies of Psychological Application, and Guangdong Key Laboratory of Mental Health and Cognitive Science, South China Normal University, Guangzhou, China

**Keywords:** emotion, distance, size, perception, grasping, visual feedback

## Abstract

Previous studies have shown that our perception of stimulus properties can be affected by the emotional nature of the stimulus. It is not clear, however, how emotions affect visually-guided actions toward objects. To address this question, we used toy rats, toy squirrels, and wooden blocks to induce negative, positive, and neutral emotions, respectively. Participants were asked to report the perceived distance and the perceived size of a target object resting on top of one of the three emotion-inducing objects; or to grasp the same target object either without visual feedback (open-loop) or with visual feedback (closed-loop) of both the target object and their grasping hand during the execution of grasping. We found that the target object was perceived closer and larger, but was grasped with a smaller grip aperture in the rat condition than in the squirrel and the wooden-block conditions when no visual feedback was available. With visual feedback present, this difference in grip aperture disappeared. These results showed that negative emotion influences both perceived size and grip aperture, but in opposite directions (larger perceived size but smaller grip aperture) and its influence on grip aperture could be corrected by visual feedback, which revealed different effects of emotion to perception and action. Our results have implications on the understanding of the relationship between perception and action in emotional condition, which showed the novel difference from previous theories.

## Introduction

Emotion-laden stimuli can affect our perception. For example, spiders appear to move faster than non-threatening objects (Witt and Sugovic, 2013); vertical distances beneath us are greatly overestimated due to fear of heights (Stefanucci and Proffitt, 2009); and threatening stimuli appear to be physically closer than non-threatening stimuli (Cole et al., 2013). Desirable objects (e.g., a bottle of water that could be used to quench one’s thirst) are often seen as much closer than less desirable objects (Balcetis and Dunning, 2010). It could be the case that such bias in perception helps regulate behaviors and promote actions (e.g., people perceive a threatening object larger in preparation to defend oneself) as proposed by previous studies (Barrett and Bar, 2009; Stefanucci and Proffitt, 2009; Balcetis and Dunning, 2010; Cole et al., 2013).

Although a lot of research has been done on how emotion influences perception, it is still unclear how the grasping taken toward an object could be influenced by the emotional state of the environment. One possibility is that the influence of emotion on action relates to and reflects its influence on perception. For example, when an object is perceived as closer as and larger than it really is, one may also reach closer and use a wider grip aperture to grasp it. If this is the case, the bias in the perception of distance and size could have serious consequences. These consequences can be seen when interacting directly with threatening stimuli, such as removing a bomb or demolition of a delicate explosive device which contains strong negative emotions (such as fear) and fine hand movements (such as grasping), simultaneously. Although the perception could be influenced, the action could usually be finished successfully.

However, since our ancestors have been dealing with threats for millions of years, it is possible that our visuomotor system has evolved and developed a way to deal with distance and size information without being influenced by any bias in perception. Indeed, it has been reported that action does not always reflect the perception of an object. For example, in the Ebbinghaus illusion, the perceived size of a target object is influenced by the size of its flankers; however, people are still able to grasp the target object according to its physical size (Aglioti et al., 1995; Haffenden and Goodale, 1998; but also see Franz et al., 2009). In addition, even when people were not able to perceive the size or shape of the target object in their periphery visual field due to the crowding effect induced by the surrounding flankers, they could still scale their grip aperture to the size or shape of the target object (Chen et al., 2015a,b). This kind of dissociation between perception and action has been considered as evidence for the influential two-visual-stream theory proposed by Goodale and Milner (1992). The theory states that there are two relatively independent visual streams: vision-for-perception mediated by the ventral stream projecting from the primary visual cortex to the occipitotemporal visual areas, and vision-for-action mediated by the dorsal stream projecting from the primary visual cortex to the posterior parietal areas (Note: this theory was also challenged by researchers. See Seegelke and Schack, 2016; Seegelke et al., 2016; and Sheth and Young, 2016). The recent evidence showing that the reweighting of sensory information varies between the perception and action systems adds additional evidence to the two-visual-systems hypothesis (Chen et al., 2018).

In this study, we conducted three experiments to test the influence of stimuli with negative and positive emotional valence on perceptual judgments and on grasping. Previous studies have used movie clips to induce emotions (e.g., Carvalho et al., 2012). But here we would like to mimic real situation that one has to grasp an object that was close to something inducing positive or negative emotions. Therefore, we prefer to use real objects as inducers. We tried other animal toys but the piloting results indicated that the toy squirrels and toy rats were most suitable for our experiment. We hypothesized that emotions may influence size estimation and grasping differently according to the two-visual-systems hypothesis.

In experiment 1, we first replicated the previous finding that emotion affects perceived distance and perceived size, but with a different way to measure the perception – that is, we asked participants to use their finger to point to where the target object was and use their fingers to manually estimate the size of the object. We did this because participants would use their fingers to perform the grasping task in experiment 2. In experiment 2, we tested how emotions influence the reaction time (RT), movement time, and grip aperture during grasping. It has been suggested that the planning and control of an action involves separate neural systems (Glover, 2004). Therefore, we also tested the influence of emotions on grasping when grasping depended solely on the planning or also on the online control of grasping. Specifically, we tested the influence of emotion on grasping in two visual feedback conditions. In the open-loop condition, participants were not able to see their hands or the target once their hands started to move away from the start button. In other words, no online adjustment based on visual feedback was available. In this case, we were able to test the influence of emotion on the planning of grasping. In the closed-loop condition, participants were able to see their hands and the target during grasping which made online adjustment based on visual feedback available. In this case, the kinematics of grasping depends on both the planning and online control of actions. We compared the influence of emotion on grasping in the open-loop and closed-loop conditions to examine the influence of emotion on the different stages of an action. In experiment 3, we repeated the operation of experiment 1 and 2 in a new group of participants to test whether participants would use the size of the toys to predict the size of the target (i.e., size-contrast effect) during size estimation or grasping. To this end, toy rats and toy squirrels used in experiments 1 and 2 were wrapped by white paper so that participants had no idea what were inside, and therefore no positive or negative emotion was induced.

## Materials and Methods

### Experiment 1: Distance and Size Estimation

#### Participants

Eighteen native students from Nanjing University (five males and 13 females, aged 19–25, and mean age=22.95), with normal or corrected-to-normal vision, took part in this experiment. The participants gave informed consent before participating. The participants had no idea of the purpose of the experiment. All the participants were right-handed, and their handedness was assessed using the Edinburgh Handedness Inventory (Oldfield, 1971). They gave written, informed consent in accordance with the procedures and protocols approved by the human subjects review committee of Nanjing University.

#### Apparatus and Stimuli

**Figure 1:**
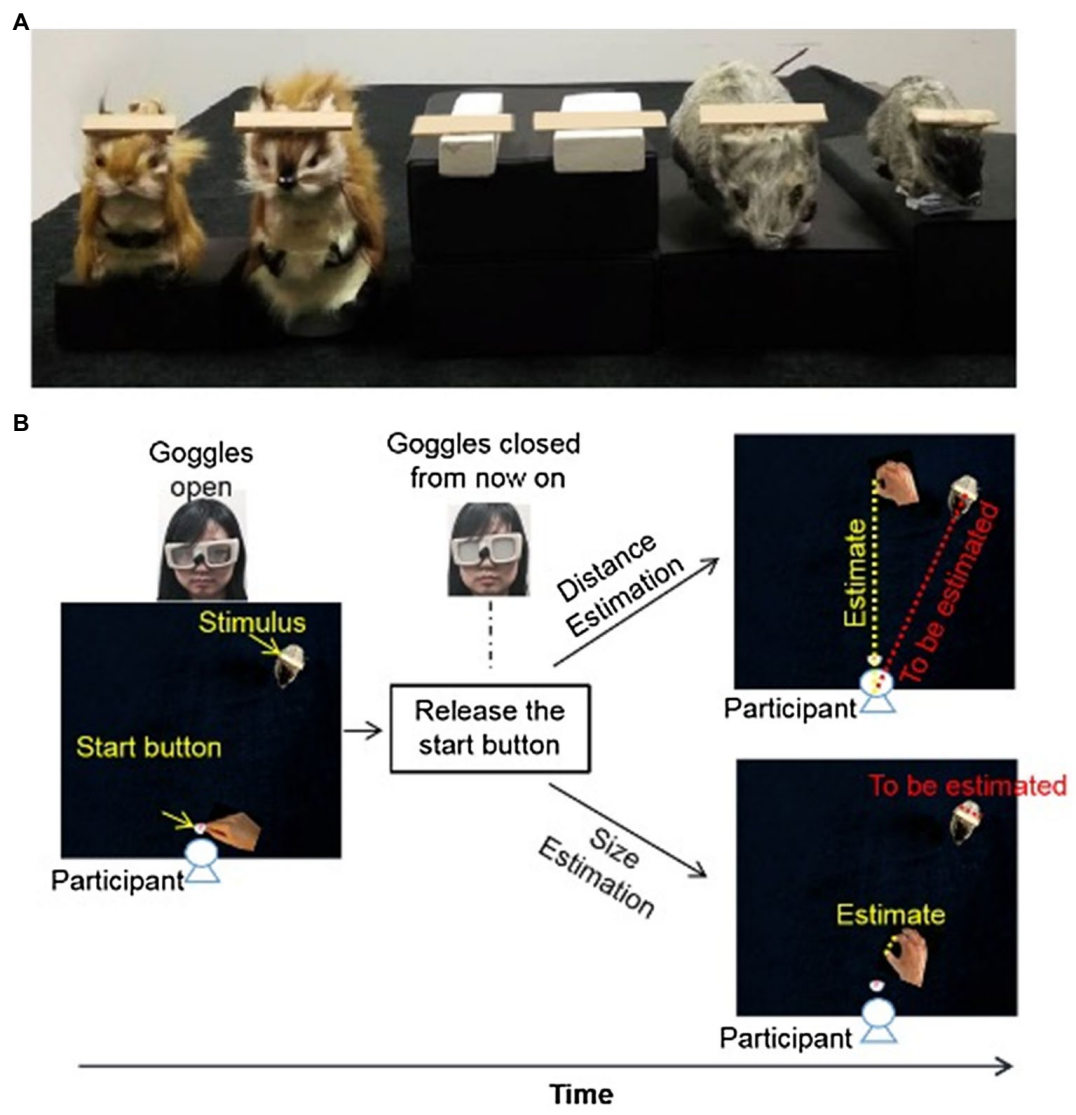
Stimuli and layout of the setup of Experiment 1. **(A)** Toy rats, toy squirrels and wooden blocks with wooden plaques (target objects) resting on them. **(B)** At the beginning of each trial, the goggles were closed. Participants were asked to hold down the start button before the task. After the experimenter opened the goggles, participants indicated the perceived distance (up) or the perceived size (bottom) of the target object. For both distance and size estimation, the goggles were closed as soon as the participants released the start button (i.e., open-loop) to prevent any online visual feedback during the estimation.

In this experiment, we examined how positive emotions (induced by brown squirrels, Figure 1A left) and negative emotions (induced by gray rats, Figure 1A right) would influence perceived distance and perceived size (Figure 1B) within the reachable space. White wooden blocks (Figure 1A middle) were used to induce neutral emotion. The target objects were rectangle wooden plaques affixed to the heads of the toys or the top surface of the wooden blocks. The gray toy rats had two different sizes (small: 10×3.5×4cm^3^, large: 14×5×6cm^3^). The brown toy squirrels also had two different sizes (small: 5×3.5×6cm^3^, large: 9×5×10cm^3^). The white wooden blocks had two different sizes (small: 10×2×1.5cm^3^, large: 10×3.5×1.5cm^3^) too. The target objects were rectangle wooden plaques of two different sizes (small: 3.5×1×0.2cm^3^, large: 5×1×0.2cm^3^) affixed to the heads of the toys or the top surface of the wooden blocks. We used inducers (toys or white wooden blocks) of two sizes to reduce the possibility of judging the size of the target object relative to the size of the inducers (i.e., size-contrast effect). This was confirmed in experiment 3 which suggests that participants would not use the size of the toys as a cue for size estimation (see results for details).

To rule out the possibility that the difference in color between the toy squirrels and the toy rats might have influenced the perceived size or perceived distance, two control conditions with brown wooden blocks (the same color with toy squirrels) and gray wooden blocks (the same color with toy rats) as objects to induce emotions were also included. The sizes of these wooden blocks were the same as the white wooden blocks mentioned above.

The toys and wooden blocks were placed at one of three distances (near: 15cm, middle: 25cm, far: 35cm) from the start button along with a line 15cm to the right of the mid-sagittal plane. Three distances were used to increase participants’ engagement in the task. The start button was located 5cm from the edge of the table facing the participants and was on the mid-line of the table. Black cubes of different heights were put under the toys and wooden blocks so that the target objects were always at the same level in all emotion conditions.

Liquid crystal goggles (PLATO goggles; Translucent Technologies, Toronto, ON, Canada) were used to control the visibility of the participant’s hands and the working space. The goggles could be switched from translucent to transparent in approximately 1ms and from transparent to translucent in 6ms or even less. The 3D positions of the thumb and index finger were recorded with an Optotrak Certus (Northern Digital, Waterloo, ON, Canada) system, with the infrared emitting diodes (IREDs) attached to the distal left corner of the index-finger nail and the distal right corner of the thumb nail. Two more IREDS were attached to the two corners of the table along the side nearest the participants to locate the center of the table, which is also the center of the participants’ body because participants were seated in the middle line of the table. The sampling rate of Optotrak was 200Hz.

#### Procedure

At the beginning of the experiment, the three target objects were presented with random sequence: the toy squirrels, toy rats, and wooden blocks. To confirm these targets induced the positive, negative, and neutral emotions, respectively, participants were asked to report how “threatened or frightened” and how “happy” they felt in the presence of each object using a Likert scale ranging from 1 (not at all) to 7 (very much). The scores of positive emotion and negative emotion were recorded, respectively.

Then, participants were asked to sit comfortably in front of the table with the mid-line of their body aligned to the middle of the table and the start button (the start button is located on the mid-line of the table). The experimenter confirmed that all participants could reach out and indicate the furthest distance of the target object comfortably without leaning forward with the thumb and index fingers of their right hand pinched together. Then participants were asked to put their chin on the chinrest throughout the testing session. They were told to fixate in a natural way and concentrated on the experimental tasks during the experiment.

At the beginning of each trial, participants were asked to hold down the start button. The goggles were closed. After the experimenter placed one of the emotion inducers and the target object on the right location, the experimenter pushed the space key to open the goggles. There were two tasks. For the perceived distance task, participants were instructed to indicate the perceived distance between the target object and themselves by reaching out and touching the table with their index finger and thumb pinched together at a point along the middle-line of the table that was perceived equally far away as the target plaque (yellow dashed line on Figure 1B for distance estimation). They were not asking to reach out along the line connecting the start button and the target plaque (red dashed line Figure 1B for distance estimation) because in that case, they might reach out until they contacted the target object.

For the size estimation task, participants were instructed to lift their fingers from the start button as soon as the googles opened, and to adjust the separation between their thumb and index finger so that the opening between the two fingers (yellow dashed line on Figure 1B for size estimation) equal to the length of the target object (red dashed line on Figure 1B for size estimation).

For both tasks, the goggles closed as soon as they released the start button so that no online visual feedback was available (i.e., open-loop). In other words, they were not able to see their hand or the target object when they were making the estimates (see Materials and Methods and Figure 1B for details).There was no time limitation for the distance and size estimation tasks. Once participants reported that they were content with their estimation, the experimenter triggered the Optotrak system to record the final positions of their fingers. Between blocks of trials (i.e., emotion conditions), participants removed the PLATO goggles and relaxed for about 5min before the next block of trials.

#### Design

A block design was used to avoid any potential interactions among trials in different emotion conditions. The toy rat blocks of trials (expected to induce negative emotion) and the toy squirrel (expected to induce positive emotion) blocks of trials were performed in a random order across participants. The order of size estimation and distance estimation was counter-balanced across participants. These two emotional blocks of trials were interleaved with the neutral blocks. There were 24 trials separately for each block, with 12 trials for each size and eight trials for each distance. The control conditions were identical with the toy rats and toy squirrel conditions except that the toys were replaced by gray and brown wooden blocks. There were eight blocks in total [4 (squirrel, rat, gray wooden block, and brown wooden block)×2 (size estimation and distance estimation)] with 24 trials in each. The control conditions and the experimental conditions were conducted in the same testing session with order balanced across participants.

#### Data and Statistical Analysis

First, to verify whether the three objects (wooden blocks, toy rats, and toy squirrels) induced the expected emotional arousal for the participants, we recorded the ratings that participants gave to each kind of inducing object separately for each of the two different sizes, and then averaged the ratings across the two different sizes. One-way repeated measures ANOVA and paired *t*-tests were used to evaluate whether the three objects induced different emotions.

To assess the effect of negative and positive emotional valence on the perception of the distance and size of objects, we extracted the estimated distance or the estimated size in each trial. The estimated distance was calculated as the distance from the midpoint between the thumb and index finger markers to the center position of the participant (i.e., the center of the two IREDS that were attached to the two corners of the table along the side nearest the participants). The estimated size was defined as the aperture between the two IREDS that were attached to the thumb and index finger when participants were indicating the perceived size (length) of the target object. Some trials were discarded due to participant’s or experimenter’s error (e.g., missing IREDs). The discard rate for estimated size was 12 of 864 (1.4%), and for estimated distance was 13 of 864 (1.5%).

Note that although the toys had different sizes and were placed at three different distances to make the task less predictable and make the participants more engaged in the task, the data of perceived distance and estimated size were averaged over the different toy sizes and different presentation distances for each emotion condition. This was because, first, participants reported the distance and size of the *target* object on top of the toys, not the toys themselves. Experiments 3 confirmed that participants would not use the relative size to judge the size of the target. Second, preliminary analysis showed that the main effect of distance was not significant.

To evaluate the effect of emotion (positive emotion induced by brown squirrels and negative emotion induced by toy rats) on perceived distance and estimated size, and to rule out of the possibility that the difference in the color of the toys might have affected the results, we performed a 2×2 repeated measures ANOVA. The first factor is color which could be brown or gray. The second factor is category which could be wooden blocks in the control conditions or the toys in the experimental conditions (i.e., four conditions in total: brown wooden blocks, gray wooden blocks, brown toy squirrels, and gray toy rats). The data in the white wooden block condition were not included for analysis because we already included the control condition with color-matched wooden blocks in the above ANVOA analysis.

#### Results

##### Emotional Arousal Scores

**Figure 2:**
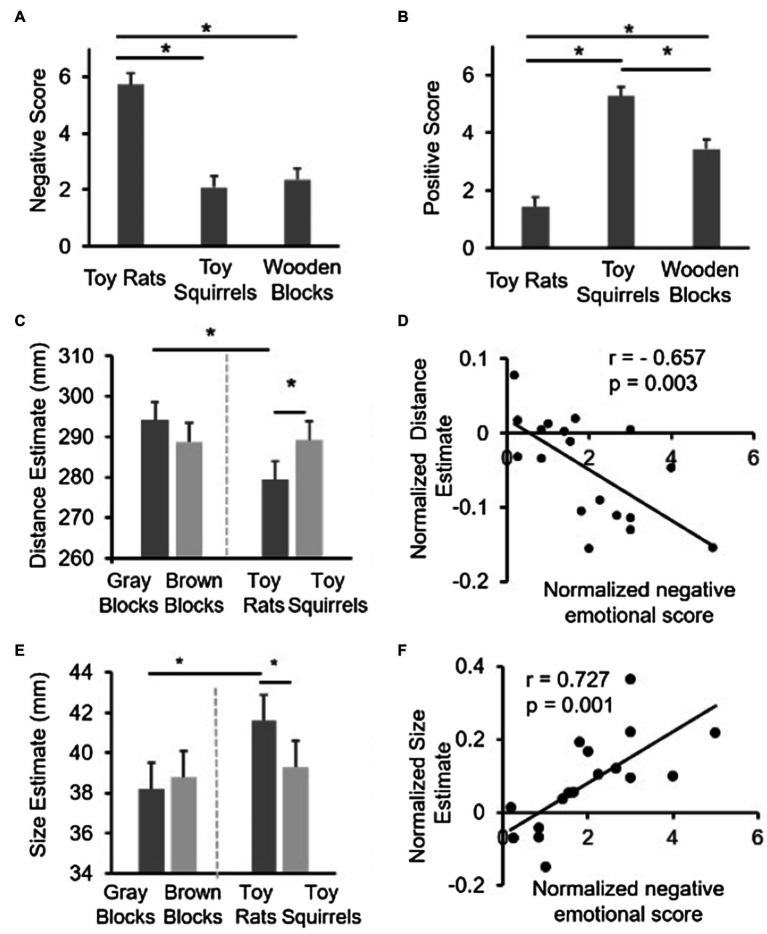
Results of Experiment 1. **(A,B)** show the data of emotional arousal scores. **(C,E)** show the size estimation and distance estimation results. Error bars represent within-subjects 95% CIs (Loftus and Masson, 1994; Masson, 2003). **(D,F)** show the correlation between the relative distance/size estimation and the relative emotion scores across participants.

One-way repeated measures ANOVAs indicated that there were significant differences among the different conditions both for the negative emotional arousal scores [*F* (2,34)=100.881, *p*<0.001] and the positive emotional arousal scores [*F* (2,34)=144.364, *p*<0.001]. Simple effect analysis based on ANOVAs revealed that, the negative emotional arousal score for the toy rats was significantly higher than those for the wooden blocks (*p*<0.001) and the toy squirrels (*p*<0.001; Figure 2A). There was no significant difference between the wooden block and the toy squirrel conditions (*p*=0.305). Thus, only toy rats induced negative emotion. On the other side, the positive emotional arousal score for the toy squirrel condition was significantly higher than the scores for the wooden blocks (*p*<0.001) and toy rats (*p*<0.001) conditions. Moreover, the scores for wooden blocks were also significantly higher than the ratings for toy rats (*p*<0.001). Thus, toy squirrels appeared to induce stronger positive emotion than both wooden blocks and toy rats (Figure 2B). The wooden blocks did not induce higher positive emotion arousal scores than the toy squirrels or higher negative emotional arousal scores than the toy rats, which suggests that when wooden object blocks were interleaved with toy rats and toy squirrel blocks, the wooden blocks could effectively prevent interactions between the toy rats and the toy squirrel blocks without introducing additional emotion effect.

##### Perceived Distance

As mentioned above, a repeated-measures ANOVA with color (brown vs. gray) and object category (wooden blocks vs. toys) was conducted to reveal the influence of color and object category on perceived distance. The interactions between color and object category [*F* (1,17)=12.079, *p*=0.003; Figure 2C] was significant, which suggested that the difference between the toy rat and toy squirrel conditions could not simply be attributed to the color difference between the two toys. The main effect of color [*F* (1,17)=0.810, *p*=0.381] and category [*F* (1,17)=3.382, *p*=0.067] was not significant. Simple effect analysis revealed that the estimated distance was significantly shorter for the toy rat condition than that for the toy squirrel condition (*p*=0.013), which is consistent with previous studies showing that people tend to feel objects associated with negative emotions closer to themselves (Cole et al., 2013). However, this was not the case for the control conditions (i.e., the gray wooden block and brown wooden block conditions; *p*=0.103) suggesting that the influence of emotion on perceived distance could not be simply attributed to the difference in color.

In addition, the distance estimation in the toy-rat condition is significantly smaller than that in the gray wooden block condition (*p*=0.008); whereas the distance estimation in the toy squirrel condition had no significant difference from that in the brown wooden block condition (*p*=0.872), which suggests that only negative, but not positive, emotion influenced the perceived distance of the target object.

To further confirm that it was the induced emotion that influenced the perceived distance of the target object, we calculated the correlation between the strength of the induced negative emotion and the changes in perceived distance across participants. Given that people may have different absolute scales for emotional rating, the emotional score in the toy-rat condition was normalized by the emotional score in the wooden-block condition to obtain a normalized emotional score [i.e., (ES_Rats_−ES_WoodenBlocks_)/ES_WoodenBlocks_, where ES indicates emotional scores]. Similarly, the normalized distance estimate was defined as (distance_Rats_−distance_GrayBlocks_)/distance_GrayBlocks_. The normalized emotional scores and the normalized distance estimate was negatively correlated (*r*=−0.657, *p*=0.003; Figure 2D; a similar correlation coefficient was calculated for the squirrel condition, but was not significant) showing that people who had stronger negative emotion on rats also perceived the target object on top of the rats closer. This provides strong evidence that it was the induced negative emotion that have influenced the perceived distance of the target object.

#### Perceived Size

Similar to the results in distance estimation, a repeated-measures ANOVA with color (brown vs. gray) and object category (blocks vs. toys) was conducted. The interactions between color (brown vs. gray) and object category [blocks vs. toys; *F* (1,17)=5.442, *p*=0.032; Figure 2E] were significant, which suggested again that the difference between the toy rat and the toy squirrel conditions could not simply be attributed to the difference in color between the two kinds of toys. The main effect of color [*F*(1,17)=0.741, *p*=0.401] was not significant while the category [*F* (1,17)=5.297, *p*=0.034] was significant. Simple effect analysis revealed that the estimated size for the target object was significantly larger when the target object was resting on top of the toy rats than when it was resting on top of the toy squirrels (*p*=0.016) suggesting that negative emotions increased the perceived size of a standard target object, which is consistent with the results of previous studies (Barrett and Bar, 2009). Importantly, the estimated size had no difference when the target object was resting on top of gray blocks compared to when it was on brown blocks (*p*=0.690).

In addition, the size estimate in the toy rat condition was significantly larger than that in the gray block condition (*p*=0.002); whereas the size estimate in the toy squirrel condition had no significant difference from that in the brown-block condition (*p*=0.669), which overall suggests that only negative, but not positive, emotion influenced the perceived size of the target object.

Similar to the correlation analysis conducted on the distance estimation task, we also performed a correlation analysis to investigate whether or not the strength of the induced negative emotion is linked to the changes in perceived size. The same normalized emotion score was used to indicate the strength of negative emotion. The normalized size estimate when the target object was resting on top of the rats was defined as (Size_Rats_−Size_GrayBlocks_)/Size_GrayBlocks_. The correlation between the normalized emotional scores and the normalized size estimate was significant (*r*=0.727, *p*=0.001; Figure 2F) suggesting that people who had stronger negative emotion on rats also perceived the target object on top of the rats larger.

### Experiment 2: Grasping

When people grasp an object, the grip aperture “in flight” is typically scaled to the size of the object (Jeannerod, 1984, 1997). In experiment 1, we found that participants perceived target objects placed on top of toy rats (negative emotion) to be larger and closer than those on top of toy squirrels (positive emotion) and wooden blocks (neutral emotion). In experiment 2, we continued to test whether the grip aperture during grasping was also similarly influenced by the negative/position emotions. We were not able to evaluate the influence of emotions in the reaching distance during grasping because participants reached the target successfully in all trials, which resulted in very small variability in reaching distance. Besides grip aperture, we also evaluated the influence of emotions on the reaction time (from the presentation of the visual stimulus to the onset of the movement of the grasping hand) of grasping and on movement time (from the onset of the movement to the stop of the movement).

**Figure 3:**
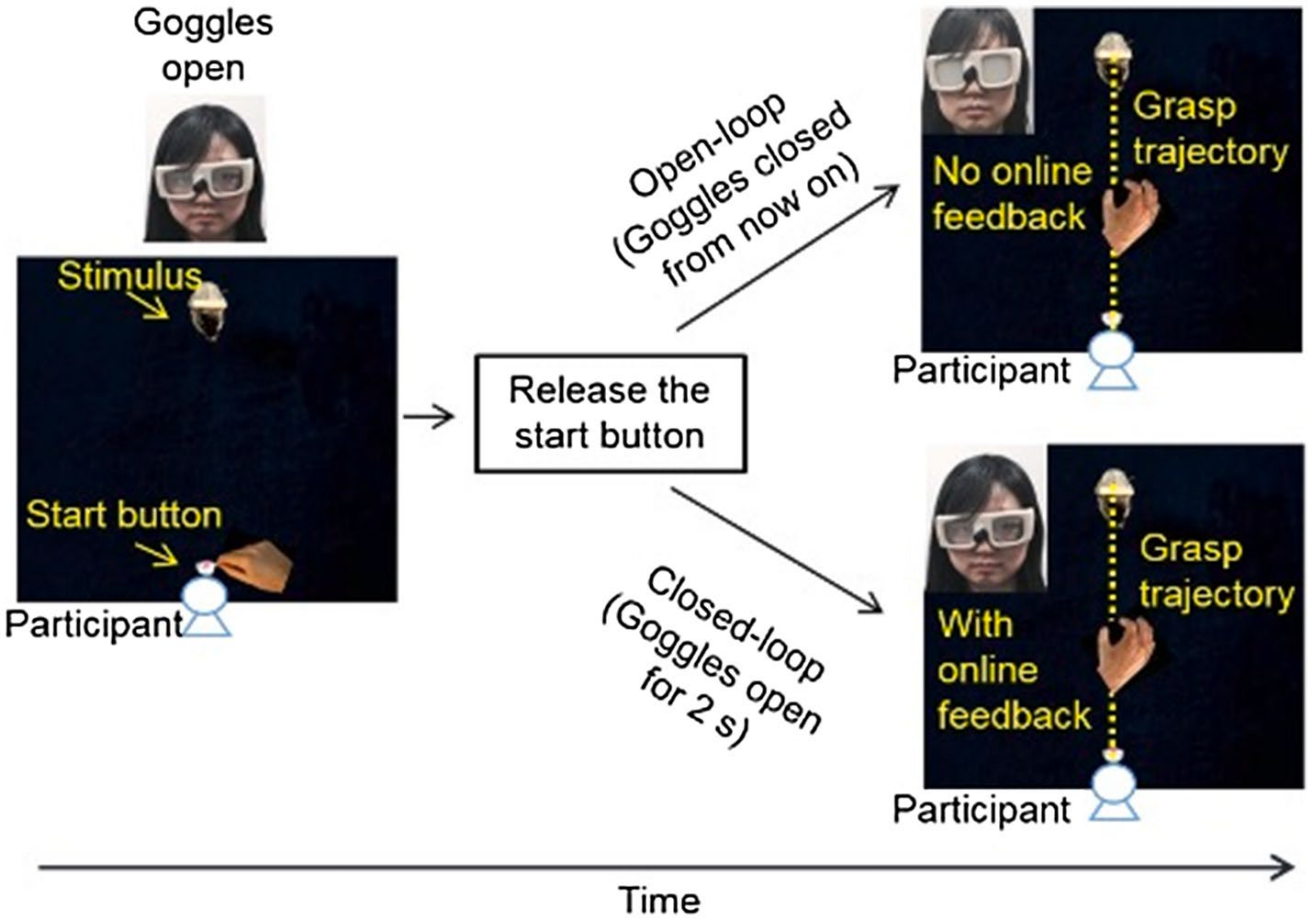
Setup and procedure for Experiment 2. Participants first held down the start button with the thumb and index finger of their right hand. The experimenter pressed a button to open the goggles when the inducer and the target object were positioned. Participants were asked to release the start button and reach out to grasp the target object along its long side naturally and accurately as soon as the goggles were opened. In the open-loop condition, the goggles were closed as soon as the start button was released, and therefore there was no online visual feedback during the execution of grasping movement. In the closed-loop condition, the goggles were open for 2s allowing a full view of the target object and the grasping hand during grasping.

Similar to experiment 1, the target object was also resting on top of toy rats or toy squirrels (experimental conditions, Figure 1A) or on gray/brown wooden blocks (control conditions). Participants were asked to reach out to pick up the target objects of different sizes at different distances with the thumb and index finger of their right hand as soon as they could view the target object. During the execution of grasping, participants’ hand and the workspace were either invisible (open-loop, goggles were closed as soon as the grasping hand released the start button) just like that in experiment 1, or were visible (closed-loop, goggles were open for 2s; Figure 3). When participant’s hand and the work space were visible during the execution of grasping movement, participants could adjust their grip aperture “in flight” based on the visual feedback during grasping, and therefore their grip aperture depends on not only the planning/programming of grasping before the execution of grasping movement but also on the visual feedback during the hand movement. In contrast, when participants were not able to see their hand or the work space during grasping, their grip aperture relied only on the programming of grasping. The difference between the results of open-loop and closed-loop conditions would indicate the role of visual feedback and online-correction in the implement of the influence of emotion on grasping.

#### Participants

Fourteen participants from experiment 1 and four new naive students from Nanjing University (five males and 13 females, aged 19–28, and mean age=23.6), with normal or corrected-to-normal vision, volunteered to take part in this experiment. The participants provided informed consent before participating. All the participants were right-handed as assessed by the Edinburgh Handedness Inventory (Oldfield, 1971). They gave written, informed consent in accordance with the procedures and protocols approved by the human subjects review committee of Nanjing University.

#### Apparatus and Stimuli

The experimental apparatus was almost identical to that used in experiment 1, except that two IREDs at the two corners of the table were removed and one more IRED was attached to the participants’ wrist to record the movement time or speed. The stimuli were identical to that in experiment 1 but were presented straight in front of the participant. We did not put the stimuli on the up-right quadrant because it would make it more difficult for the participants to reach to the correct position especially in the open-loop condition when participants were not able to see the stimuli or their grasping hand during grasping. The apparatus and stimuli of the control conditions (i.e., gray and brown wooden block conditions) were identical to that in the experimental conditions (i.e., rat and squirrel conditions).

#### Procedure

As in experiment 1, at the beginning of the experiment, participants were also required to report their feeling of the toy rats, toy squirrels, and wooden blocks using a Likert scale ranging from 1 (not at all) to 7 (very much).

In the main experiment, participants were seated comfortably at the table with their mid-line aligned with the start button. Participants were positioned so that that could grasp the target object comfortably without leaning forward even when the target object was positioned at the furthest distance. The participants were asked to put their chin on the chinrest throughout the testing session.

At the beginning of each trial, participants held down the start button with their thumb and index finger pinched together. The goggles were closed. After the experimenter placed the target object and the toy rat/squirrel or the wooden block in the right location, she hit a button to open the goggles and trigger the Optotrak to record the positions of the IREDs.

Participants were asked to reach out and grasp the target object with their thumb and index finger at a natural pace, and to lift it up and put it down. In open-loop condition, the lenses of the goggles became opaque as soon as the participant released the start button (same as in experiment 1). In the closed-loop condition, the lenses of the goggles remained transparent for 2s following the participant’s release of the start button, permitting a full view of the hand and the object during the execution of grasping movement (Figure 3). Participants were told that all trials would be either closed-loop or open-loop in the same block. Between blocks, participants removed the PLATO goggles and relaxed for 5min before beginning the next block of trials.

#### Design

Again, the different emotion conditions used in experiment 2 were also run in separate blocks. The neutral condition (i.e., wooden block condition) was always interleaved with the negative or positive condition to avoid potential interactions between the negative and positive emotional blocks. The order of the negative and positive emotion conditions was counter-balanced across participants. Participants were required to perform their grasping movements separately for the two “feedback” conditions (closed-loop vs. open-loop), and the order was also counter-balanced across the participants. Therefore, there were eight blocks in total [2 feedback conditions×(2 neutral+1 positive+1 negative) emotion conditions] with 24 trials in each.

#### Data and Statistical Analysis

First, we analyzed the ratings that participants gave for each kind of object for each of the two different sizes. One-way repeated measures ANOVAs and paired *t*-tests were used to examine whether the toys induced different emotions.

Then to assess the effect of negative and positive emotional valence on the grasping behavior, we extracted the RT, movement time, and peak grip aperture (PGA) from each grasping trial. The RT was defined as the time interval between the goggles were opened and the movement onset of wrist IRED. The movement onset was defined as the first frame of the 40 consecutive frames during which the wrist IRED exceeded 20mm/s, the movement offset was defined as the first frame of the 40 consecutive frames during which the wrist IRED fell below 20mm/s. The movement time was defined as the time between movement onset of wrist IRED and the movement offset of wrist IRED. Participants’ thumb and index fingers were pinched together before grasping. When they reached out to grasp an object, the aperture between the two fingers first opened, reached to peak well before they made contact with the target object, and then closed down to hold the object (see Chen et al., 2015a; for a full profile of grip aperture during grasping). The PGA was defined as the maximum vector distance between the index-finger and thumb IREDs that was achieved from the start of the movement to when the fingers contacted the target object. Therefore, PGA always happens before participants contact the target object, and is commonly used in grasping studies to indicate whether or not grip aperture is correlated to the size of the object (Castiello, 2005). The search window of PGA terminated at the movement offset of wrist IRED.

Some trials were excluded due to participant or experimenter error (e.g., missing IREDs). The discard rate for RT was 84 of 3,456 (2.4%), and for PGA was 28 of 3,456 (0.8%). Repeated-measures ANOVAs with three factors {[2 visual feedback (closed-loop vs. open-loop)×2 colors (gray vs. brown)×2 categories (toy vs. block)]} were conducted to reveal the influence of color and object category on reaction time and PGA.

#### Results

##### Emotional Arousal Scores

The result is consistent with the emotional score result in experiment 1. One-way repeated measures ANOVA indicated that there were significant differences among different emotions both for the negative [*F* (2,34)=84.944, *p*<0.001] and positive emotional arousal scores [*F* (2,34)=64.170, *p*<0.001]. The negative emotional arousal score for the toy rat condition was significantly higher than those for the wooden block condition (*p*<0.001) and the toy squirrel condition (*p*<0.001). There was no significant difference in the negative arousal scores between the wooden block and toy squirrel conditions (*p*=0.886). The positive emotional arousal score in the toy-squirrel condition was significantly higher than that in the wooden-block condition (*p*<0.001) and toy-rat condition (*p*<0.001). Moreover, the positive emotional score for the wooden block condition was also significantly higher than the score for the toy rat condition (*p*<0.001). In sum, toy rats and toy squirrels induced negative emotion and positive emotion, respectively.

##### Reaction Time

**Figure 4:**
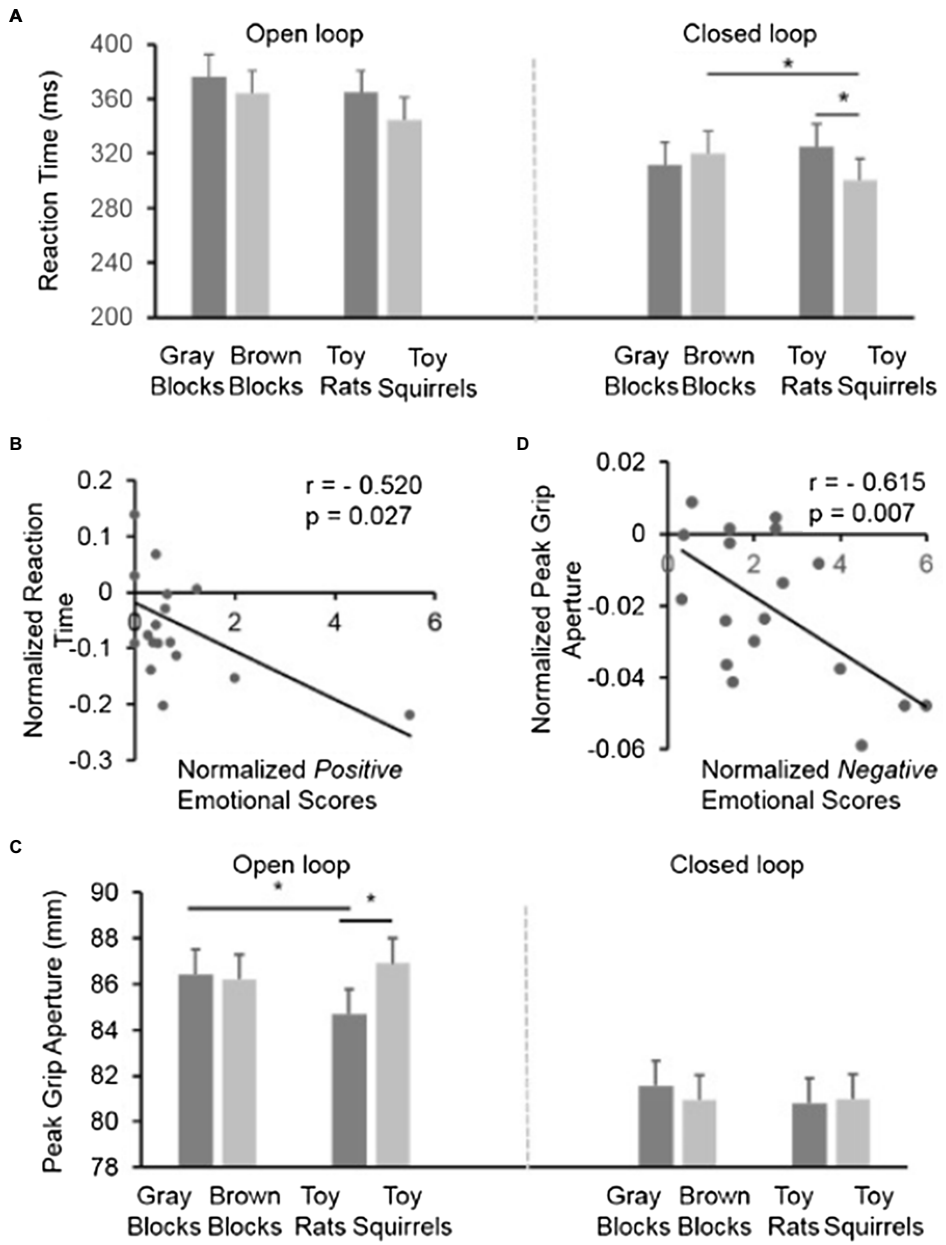
The results in Experiment 2. **(A)** The reaction time in the control conditions (gray blocks and brown blocks) and the experimental conditions (toy rats and toy squirrels) when participants had no visual feedback (open-loop) or had visual feedback (closed-loop) during the execution of grasping. **(B)** The correlation between the normalized positive emotional scores and the normalized reaction time in the closed-loop condition across individuals. Note, although there seems an outlier, the correlation was still strong after the “outlier” was removed. In other words, the correlation could not be attributed to the existence of the “outlier.” **(C)** The peak grip apertures (PGAs) during grasping in the control conditions (gray blocks and brown blocks) and the experimental conditions (toy rats and toy squirrels) when participants had no visual feedback (open-loop) or had visual feedback (closed-loop) during the execution of grasping. **(D)** The correlation between the normalized negative emotional scores and the normalized peak grip aperture in the open-loop condition across individuals.

As shown in Figure 4A, the reaction time in the open-loop condition when participants were not able to see the target object and their grasping hand during grasping (i.e., without visual feedback) was overall longer than the reaction time in closed-loop when the target object and the grasping hand were both visible. This was confirmed by a significant main effect of visual feedback on the reaction time [*F* (1,17)=6.282, *p*=0.023] when a three-way repeated-measures ANOVA [2 visual feedback (closed-loop vs. open-loop)×2 colors (gray vs. brown)×2 categories (toy vs. block)] was conducted. The interaction of three-way repeated-measures ANOVA was not significant [*F* (1,17)=1.210, *p*=0.287]. The interaction between colors and visual feedback was not significant [*F* (1,17)=0.552, *p*=0.468], and neither was the interaction between categories and visual feedback [*F* (1,17)=1.336, *p*=0.264]. The main effect of categories [*F* (1,17)=4.479, *p*=0.049] was significant while colors[*F* (1,17)=3.672, *p*=0.072] was not significant. The interaction between colors and categories [*F* (1,17)=4.157, *p*=0.057] was marginally significant suggesting that it was not just the color of the toys that modulated the reaction time.

To have a close look at the influence of the toy rats and toy squirrels on the reaction time, we performed a simple effect test to reveal any difference in the effect of emotion on reaction time separately for the open- and closed-loop conditions. In the open-loop condition when participants were not able to see the target or the grasping hand during grasping, there was no significant difference in the reaction times between the toy-rat or toy-squirrel conditions (*p*=0.116). Moreover, the small (and non-significant difference) in reaction time between the toy-rat and the toy-squirrel conditions was as small as that between the gray-wooden block and the brown-wooden block conditions, which suggests that this small difference may be explained by the difference in color.

In contrast, in the closed-loop condition, when participants were able to see the target object and their grasping hand, the reaction time in grasping the target object was shorter when the target object was resting on top of the toy squirrels than when it was resting on top of the toy rats (*p*=0.007) and brown wooden blocks (*p*=0.045). The reaction time had no difference in the toy rat and the gray block conditions (*p*=0.148) suggesting that the negative emotion induced by toy rats did not influence the reaction time. The reaction time in the toy squirrel condition was significantly smaller than that in the brown blocks condition suggesting that people tended to grasp the object on top of an object that induces positive emotion faster. Overall, the results suggest that visual feedback is helpful for the implement of the modulation of positive emotion.

Similarly, we also calculated the correlation between the normalized emotional scores (ES) and the normalized RT when the target object was on the squirrels in the closed-loop condition. In the toy rat condition, we calculated correlation between the value of (ES_Rat_−ES_WoodenBlocks_)/ES_WoodenBlocks_ and (RT_Rat_−RT_BrownBlocks_)/RT_BrownBlocks_. The correlation coefficient was not significant (*r* =0.001, *p*=0.997). In the toy squirrel condition, we calculated correlation between the value of (ES_Squirrel_−ES_WoodenBlocks_)/ES_WoodenBlocks_ and (RT_Squirrel_−RT_BrownBlocks_)/RT_BrownBlocks_. The correlation between the normalized emotional scores and the normalized reaction time was negative and significant (*r*=−0.520, *p*=0.027) suggesting that people who had stronger positive emotion on the inducer also had shorter reaction time when reached out to grasp the object on it (Figure 4B).

##### Peak Grip Aperture

It could be clearly seen in Figure 4C that the PGAs in the closed-loop condition were overall much smaller than the PGAs in the open-loop condition. This is confirmed by a significant main effect of visual feedback [i.e., open- vs. closed-loop; *F* (1,17)=22.504, *p*<0.001)] when a three-way repeated-measures ANOVA [2 visual feedback (closed-loop vs. open-loop)×2 colors (gray vs. brown)×2 categories (toy vs. block)] was conducted. The larger PGA in the open-loop condition is in line with previous results (Tang et al., 2014, 2015; Chen et al., 2015a) and could be due to the uncertainty of grasping in the open-loop condition when participants were not able to see their hand and the target object during the execution of grasping. The three-way interaction of visual feedback, colors, and categories was not significant [*F* (1,17)=1.980, *p*=0.177]. The interaction between colors and visual feedback was significant [*F* (1,17)=8.123, *p*=0.011], but the interaction between categories and visual feedback [*F* (1,17)=0.051, *p*=0.825] was not, neither was the interaction between categories and colors [*F* (1,17)=3.655, *p*=0.073]. The main effect of categories [*F* (1,17)=0.676, *p*=0.422] and colors [*F* (1,17)=1.598, *p*=0.223] was not significant.

To have a closer look at the effect of emotion when visual feedback was available or unavailable, we performed repeated measures ANOVAs [2 colors (gray vs. brown)×2 categories (toy vs. block)] for the closed-loop and open-loop conditions separately. In the open-loop condition (i.e., the target object and the grasping hand were invisible), the interaction of color×category is significant [*F* (1,17)=8.541, *p*=0.009] suggesting that the influence of toys on PGAs is different from the influence of the color of the blocks on PGAs. The simple main effect analysis revealed that participants opened their thumb and index finger smaller when the target object was on top of the toy rats than it was on top of the toy squirrels [*F* (1,17)=17.26, *p*=0.001] or gray blocks [*F* (1,17)=17.9, *p*=0.001]. But the grip aperture on the toy squirrel condition did not differ from the grip aperture for grasping the same target in the brown block condition [*F* (1,17)=0.6, *p*=0.447]. And the grip aperture on the gray block did not differ from the grip aperture for grasping the same target on the brown block condition [*F* (1,17)=0.11, *p*=0.746]. In the closed-loop condition, neither the main effects of color or category [both *F* (1,17)<0.647, *p*>0.432], nor the interaction between color and category [*F* (1,17) =0.455, *p*=0.509] was significant when the target object and grasping were visible and thus adjustment of grip aperture was available.

Overall, the grip aperture was only influenced by the negative emotion induced by toy rats but not by the positive emotion induced by squirrels and the influence happened only when participants were not able to see the target or their grasping hand (i.e., open-loop when visual feedback was not available). In other words, the visual feedback during grasping in the closed-loop condition did not only decrease the overall grip aperture during grasping but also eliminated the influence of negative emotion on grip aperture.

Similarly, we also calculated the correlation between the normalized emotional scores [i.e., (ES_Rats_−ES_WoodenBlocks_)/ES_WoodenBlocks_, where ES indicates emotional scores] and the normalized PGA [i.e., PGA_Rats_−PGA_BrownBlocks_)/PGA_BrownBlocks_] when the target object was on the rat in the open-loop condition (we also calculated the correlation in the toy squirrel condition, but the correlation coefficients was not significant). The correlation was significant [*r*=−0.615, *p*=0.007] suggesting that the stronger the induced negative emotion, the smaller the grip aperture during grasping (Figure 4D).

Moreover, to directly compare whether the induced emotions by toy rats and toy squirrels had different effects on size estimation (experiment 1) and PGA (experiment 2 in open-loop condition), we performed a two-way repeated-measures ANOVA with emotion (two levels: toy rats vs. toy squirrels) and task measurements (two levels: size estimation in perception task vs. PGA in grasping task) as factors. Note: only the data of participants who took part in both experiments were included. We found that the interaction between the two factors was significant [*F* (1,13)=20.633, *p*=0.001]. Simple effect analysis revealed that, the PGA was significantly different from the size estimation in positive emotion condition [(1,13)=18.64, *p*=0.001] and also in negative emotion [*F* (1,13)=5.59, *p*=0.034]. It indicates that the emotions (rats vs. squirrels) influenced the size estimation and PGA differently, although PGA was much larger than the size estimation.

##### Movement Time

Movement time is also an important kinematic feature of action. A three-way repeated measures ANOVA [2 categories (blocks vs. toys)×2 colors (gray vs. brown)×2 visual feedback (open-loop vs. closed-loop)] was performed. The three way interaction was not significant [*F* (1,17)=2.859, *p*=0.109]. The interaction between colors and visual feedback was not significant [*F* (1,17)=0.856, *p*=0.368], neither was the interaction between categories and visual feedback [*F* (1,17)=0.745, *p*=0.4]. In addition, the interaction between categories and colors was not significant [*F* (1,17)=0.978, *p*=0.337]. The main effect of visual feedback [*F* (1,17)=2.007, *p*=0.175] and colors [*F* (1,17)=0.004, *p*=0.949] was not significant, while the main effect of categories was significant [*F* (1,17)=4.448, *p*=0.05], the movement time in toys condition (*M*=934.205, *SE*=23.685) was longer than the movement time in blocks condition (*M*=899.775, *SE*=25.957), but no significant effects of colors and visual feedback were found which may because the participants were required to grasp the objects in nature pace.

### Experiment 3: Size Estimation and Grasping in Neutral Emotion Condition

In experiments 1 and 2, we pooled the data from toys of two sizes. We used two sizes aiming to reduce the possibility that participants would use the relative size of the target to the toys (emotion inducers) as cues to perform size estimation and grasping task. To confirm this, we performed a third study in which the toys were wrapped tightly by a piece of white paper. In this case, the size of the toys was the same as before but no emotions would be induced. Participants performed the same size estimation and grasping tasks as experiments 1 and 2.

#### Participants

Twenty six naive students from Nanjing University (14 males and 12 females, aged 19–28, and mean age=24), with normal or corrected-to-normal vision, volunteered to take part in this experiment. Four participants accomplished the wrapped squirrels condition but quit the wrapped toy rats condition for personal reasons. The participants provided informed consent before participating. All the participants were right-handed as assessed by the Edinburgh Handedness Inventory (Oldfield, 1971). They gave written, informed consent in accordance with the procedures and protocols approved by the human subjects review committee of Nanjing University.

#### Apparatus, Stimuli, and Procedure

The experimental apparatuses, the target objects, and the procedures of size estimation trial and grasping trial were identical to those in experiment 1 and experiment 2, respectively, in the perception task and grasping task. The only difference in experiment 3 was that the toy rats and squirrels were wrapped by white paper to eliminate the color effect and emotion effect. We tested in this neutral emotion case, whether the size estimation and grasping of the target would be affected by the size of the toys.

#### Design

In each of the three tasks (size estimation task, open-loop grasping, and closed-loop grasping task), there were four conditions [2 inducer size (small, large)×2 target object size (small, large)] for each kind of wrapped toy. For each tasks, there were four blocks of 24 trials, with six trials for each condition. Each task was carried out for two times, one for the wrapped toy rats and the other one for the wrapped toy squirrels. The order of the different target object size and different neutral stimuli size conditions was counter-balanced across participants. The order of wrapped toys type was counter-balanced across participants. Participants were required to perform their grasping movements separately for the two feedback conditions (closed-loop vs. open-loop), and the order was also counter-balanced across the participants.

#### Data and Statistical Analysis

The same methods were used to extract the size estimation and PGA values in each trial. To exclude the effect of extra variables (e.g., shape of toys and size of toys) on size estimation and PGA, three 3-way repeated measures ANOVAs were performed with the type of wrapped toys (wrapped toy rats and wrapped toy squirrels), toy size and target size on the size estimation, and PGA in open loop and closed loop separately.

#### Results

**Figure 5:**
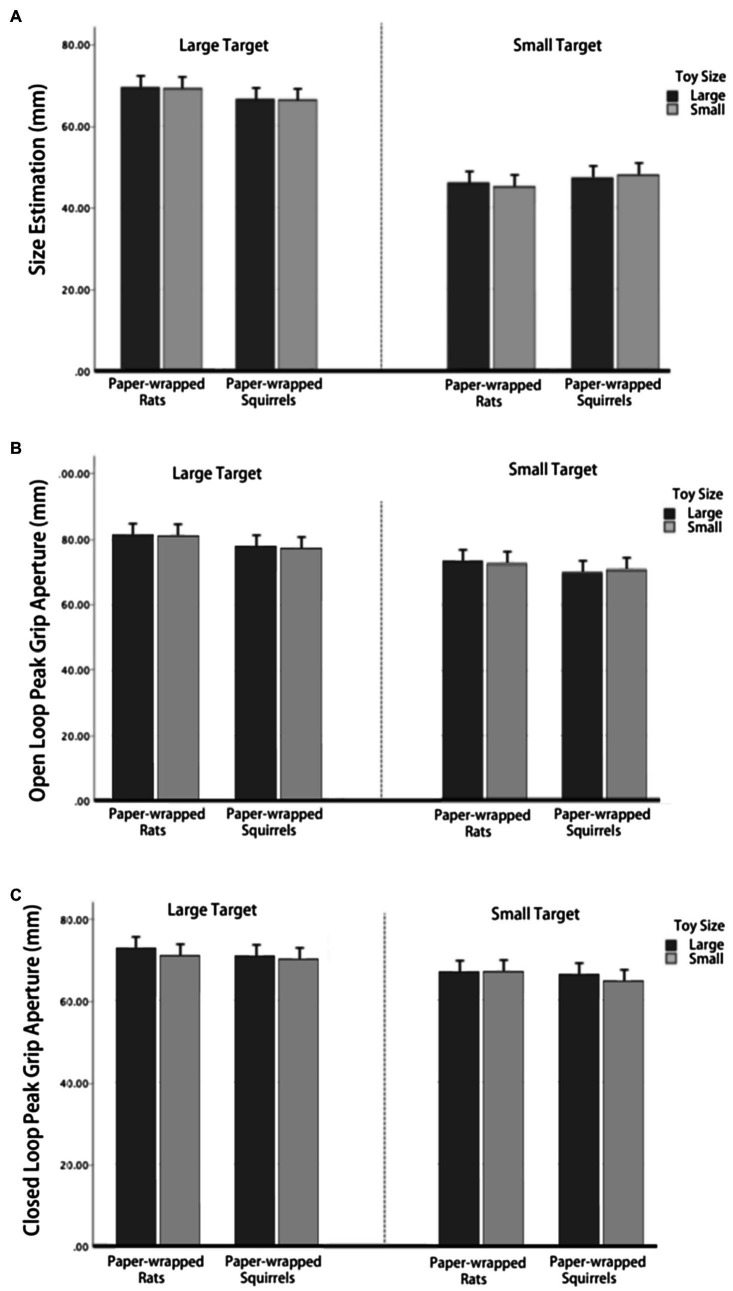
Results of Experiment 3. **(A)** Size estimation when paper-wrapped toys were used as inducers. **(B)** Peak grip aperture when paper-wrapped toys were used as inducers in open-loop condition. **(C)** Peak grip aperture when paper-wrapped toys were used as inducers in closed-loop condition.

As shown in Figure 5A, in size estimation task, neither the three-way interaction was not significant [*F* (1,21)=9.89, *p*=0.331], nor the interaction between toy types and toy size [*F* (1,21)=1.252, *p*=0.276], toy size and target size [*F* (1,21)=0.026, *p*=0.874], or toy types and target size [*F* (1,21)=32.92, *p*=0.084]. The main effect of toy size [*F* (1,21)=0.080, *p*=0.781] and toy types [*F* (1,21)=0.065, *p*=0.801] was not significant. The main effect of target object size was significant [*F* (1,21)=111.173, *p*<0.001], and size estimation was larger in large target condition. As shown in Figure 5B, in the open loop condition, the three-way interaction was not significant [*F* (1,21)=1.478, *p*=0.238], nor the interaction between toy types and toy size [*F* (1,21)=0.593, *p*=0.450], toy size and target size [*F* (1,21)=0.462, *p*=0.504], or toy types and target size [*F* (1,21)=0.174, *p*=0.681]. The main effect of toy size [*F* (1,21)=0.194, *p*=0.664] and toy types [*F* (1,21)=2.922, *p*=0.102] was not significant. The main effect of target object size was significant [*F* (1,21)=33.110, *p*<0.001], size estimation was larger in large target condition. As shown in Figure 5C, in the closed loop condition, the three-way interaction was not significant [*F* (1,21)=2.176, *p*=0.155], nor the interaction between toy types and toy size [*F* (1,21)=0.114, *p*=0.739], toy size and target size [*F* (1,21)=0.274, *p*=0.606], or toy types and target size [*F* (1,21)=0.001, *p*=0.990]. The main effect of target size was significant [*F* (1,21)=43.905, *p*<0.001], size estimation was larger in large target condition. The main effect of toy types was not significant [*F* (1,21)=2.084, *p*=0.164], and the toy size was not significant [*F* (1,21)=3.672, *p*=0.069].

These results suggested that in these three conditions, the shape and size of neutral stimuli did not have an effect on the perception or the PGA, only the target object size would affect the estimation and grasping which excluded the uncertainty of previous results for the possible effect of stimuli size and shape. Therefore, we thought we offered an available verification for this study.

## Discussion

In this study, we first carried out two experiments to investigate how emotional stimuli (toy rats and toy squirrels) affected the distance and size perception of a target object resting on them and the actions directed toward that target object. The results of Likert scales confirmed that the toy rats induced negative emotions while the toy squirrels induced positive emotions. In experiment 1, we found that participants perceived the target object on top of the toy rats (induced negative stimulus) significantly closer and larger than the same target object resting on top of the toy squirrels (induced positive stimulus) suggesting that there is perceptual bias even within the reachable personal space. In experiment 2, when no visual feedback was available (i.e., open-loop condition), participants used a smaller grip aperture to grasp the target object when it was placed on top of the toy rats (negative emotion) than when it was placed on top of the toy squirrels (positive emotion). The different effects of negative emotion on size perception and grip aperture (bigger size perception but smaller grip aperture) suggest that there are independent neural mechanisms for the influence of negative emotion on perception and action. In addition to the influence of negative emotion on grip aperture, we also found that positive emotion sped up the onset of the grasping movement (i.e., reaction time) but only when visual feedback was available (i.e., closed-loop condition). Moreover, the effects of negative emotion on the size and distance perception and grip aperture, along with the effects of positive emotions on the reaction time of grasping, as addressed above, were correlated with the strength of the corresponding emotions across individuals. It may further indicate that the induced emotion caused the observed changes in perception or action. At last, we also performed a control experiment which showed that the effects of induced emotions by toy rats and toy squirrels on the perception and grasping of targets were not affected by the shape of toy rats and toy squirrels in our study.

### Influence of Negative Emotion on Perception

To exclude the possibility that participants exploited the clues of their limbs and surrounding items to estimate the target distance, the target location in distance estimation task was designed to be different with the size estimation task and grasping task. With wooden blocks of the same color used as controls, we found that only the toy rat (not the toy squirrels) which induced negative emotions affected the perceived size and perceived distance of the target object. Majority of previous studies that examined the influence of emotion on perception tested whether the perception of the threatening objects themselves was biased (van Ulzen et al., 2008; Cole et al., 2013). In our experiment, we go beyond these studies by showing that the perception of a neutral target object could be biased when it was attached to (i.e., on top of) an object that induces positive/negative emotions. Moreover, we showed that this bias occurs even in the reachable peri-personal space where visual acuity is relatively high.

How do we then explain the influence of negative emotion on size and distance perception? One possibility is that emotional valence affects perception in ways that reflect heightened attention. For example, coins seemed to be larger than same-size cardboard disks for children from a poor family (Bruner and Goodman, 1947). It might also be adaptive to perceive a threatening stimulus (such as a rat) as both closer and larger than really is (Barkow et al., 1995).

There is evidence showing that the perceived size of an object is represented in the primary visual cortex (Sperandio et al., 2012). Additionally, it has been long known that the negative emotion is related to the amygdala (Pessoa, 2008). Based on this, we speculate that the influence of negative emotion on size perception must involve interactions between the amygdala and the primary visual cortex or interactions between the amygdala and the visual signals in the subcortical visual structures before they arrive at the primary visual cortex for initial processing. Further studies are required to explain the dynamics of the possible interactions.

### Influence of Emotion on Action

First, the reaction time (defined as the interval between when the participants were allowed to see the object and when their hand started to move) was shorter for the squirrel condition when visual feedback was available (i.e., closed-loop condition). These results are consistent with those of a large number of studies that have demonstrated differences in the reaction times for approach and avoidance when participants are confronted with stimuli of different emotional valences (Solarz, 1960; Chen and Bargh, 1999; Neumann and Strack, 2000; Wentura et al., 2000; Duckworth et al., 2002; Seibt et al., 2008). For example, Chen and Bargh (1999) found participants were faster to respond to negative emotional valence stimuli when pushing the lever away (avoid) than when pulling it toward them (approach) but were faster to respond to positive stimuli by pulling than by pushing the lever; Duckworth et al. (2002) found that novel stimuli produces muscular predispositions. Going beyond the previous studies, our study shows that a shorter reaction time was observed only in the closed-loop condition, but not the open-loop condition, suggesting that the effect of positive emotion may only occur at the execution (not the planning) stage of grasping.

Second, the PGA was smaller when the target object was on top of a toy rat than when it was on top of a toy squirrel and its corresponding controls (gray blocks) when visual feedback was not available (i.e., open-loop condition). This suggests that participants tend to open their hand less when the target object was associated with negative emotions. The smaller grip aperture probably reflects the fact that participants were unwilling to grasp the target object that was associated with negative emotion or were trying to avoid the unpleasant toy rat during grasping. However, the influence in grip aperture disappeared in the closed-loop condition suggesting that the online visual feedback and adjustment could eliminate the influence of negative emotion on the programming stage (i.e., open-loop condition). The different effects observed in the open and closed-loop condition provide further evidence that the planning and control of an action involves different neural network (Glover, 2004).

### Perception vs. Action

In our study, we showed that the influence of negative emotion on perception and action is independent: when the target object was positioned on top of the rats, participants perceived the target object as larger than when it was on top of the squirrels, but used a smaller grip aperture to grasp it.

Although it is unclear why the difference in the effect of emotion on perception and action was, our results seems to be consistent with the two-visual-stream theory (Goodale and Milner, 1992) which states that there are two relatively independent (though also interactive) visual streams: the visual ventral stream mediating vision for perception and the visual dorsal stream mediating vision for action.

Although there was evidence showing that action is less susceptible to the influence of objects in the surroundings (Aglioti et al., 1995; Haffenden and Goodale, 1998; Chen et al., 2015a,b), it is important to note that grasping does not always escape the influence of the surroundings that bias the perception of the target object. According to the two-visual-streams theory, if the influence of the surroundings happens before or during the initial signal processing in the primary visual cortex, then the consequence of this influence will be passed on to both visual systems. As a result, both perception and action will be influenced. For example, tilt illusion influences both perception and action but the Rod and Frame illusion only influences perception but not action (Milner and Dyde, 2003). Previous studies have shown that negative emotion is mediated by subcortical structures such as the amygdala (Sander et al., 2003), which are close to subcortical visual structures such as lateral genicular nucleus (LGN) and superior sulcus (SC; Stefanacci and Amaral, 2000; Stefanacci and Amaral, 2002). Therefore, it is possible that the negative emotional signal in the amygdala has modulated the visual signal in LGN or SC, and as a result both perception and action are influenced by the negative emotion in open-loop condition.

### Negative vs. Positive Emotions

The grasping in the open-loop condition depends only on the programming of grasping based on the visual information participants obtained before the hand movement. Conversely, the grasping in the closed-loop condition depends on both programming before the hand movement (planning stage of grasping) and adjustment based on visual feedback during hand movement (execution stage of grasping). Our finding that the negative emotion influences grip aperture only in the open-loop condition but positive emotion influences reaction time only in the closed-loop condition revealed a critical difference between negative and positive emotions.

It appears that the influence of negative emotion is automatic and pre-attentive, and therefore appears during the programming of grasping in the open-loop condition, but could be eliminated by visual feedback in the closed-loop condition. In contrast, the influence of positive emotion on reaction time is gradually accumulated during the consecutive closed-loop trials with visual feedback. We speculate that the influence of positive emotion on reaction time occurs gradually because visual feedback on a particular trial was received after participants released the start button, i.e., after the reaction time was measured. Therefore, it must be feedback from previous trials in the same closed-loop block that influenced the reaction time of the upcoming trial(s), which resulted in a reduction in the mean reaction time of all trials.

## Conclusion

To conclude, we found that when a target object was associated with negative emotion, the target object would be perceived as larger and closer, but participants would use a smaller grip aperture to grasp the target object. In addition, the smaller grip aperture during grasping was observed when participants were not allowed to see the target or their hand during the execution of grasping but was not observed when the target and their hand were visible during grasping. Furthermore, positive emotion only influences the reaction time to object grasping when participants could see the target and their hand. Our results have implications on the understanding of the relationship between perception and action in emotional condition, which showed the novel difference from previous theories.

## Data Availability Statement

The raw data supporting the conclusions of this article will be made available by the authors, without undue reservation.

## Ethics Statement

The studies involving human participants were reviewed and approved by Ethics Committee at Nanjing University. The patients/participants provided their written informed consent to participate in this study. Written informed consent was obtained from the individual(s) for the publication of any potentially identifiable images or data included in this article.

## Author Contributions

CS designed and conducted the experiment, analyzed the data, and wrote the article. JC wrote and revised the article. YC designed and conducted the experiment and analyzed the data. RT designed the experiment, wrote, and revised the article. All authors contributed to the article and approved the submitted version.

## Funding

This study was supported by grants from National Natural Science Foundation of China (Grant #31571131 to RT and No. 31800908 and No. 31970981 to JC), the fourth pilot research program for human spaceflight (030602) to RT, and the Key Realm R&D Program of Guangzhou (202007030005) to JC.

## Conflict of Interest

The authors declare that the research was conducted in the absence of any commercial or financial relationships that could be construed as a potential conflict of interest.

## Publisher’s Note

All claims expressed in this article are solely those of the authors and do not necessarily represent those of their affiliated organizations, or those of the publisher, the editors and the reviewers. Any product that may be evaluated in this article, or claim that may be made by its manufacturer, is not guaranteed or endorsed by the publisher.
